# Skeleton Notes on Cataphoresis

**Published:** 1896-12

**Authors:** Louis Jack


### SKELETON NOTES ON CATAPHORESIS.[1]

BY DR. LOUIS JACK.

THE apparatus in all but one case as noted was the same as employed in the clinic. The millii given is the reading of the current through the dentine.

CASE I.—H. H. F.; proximate of second superior bicuspid; cocaine hydrochloride; ten cells; forty-second pin; four-tenths millii; thirteen minutes; relief complete; pulp found to be exposed.[2]

CASE II.—N. M.; cocaine hydrochloride; fifteen cells; seventieth pin; eleven minutes; second application; fifteen cells; eightieth pin; two-tenths millii; relief complete.

CASE III.—R. M.; left superior third molar; cocaine hydrochlor. eighteen per cent.; fifteen cells; thirty-eighth pin; thirteen minutes; relief complete.

CASE IV.—C. M.; cocaine citrate; ten cells; forty-fifth pin; relief nearly complete.

CASE V.—G. H. S.; two proximate cavities of lateral and cuspid, caused by caries at the cervical margin of gold fillings. The broad

---

[1] Read before the Academy of Stomatology, Philadelphia, October 27, 1896.

[2] There is a striking feature regarding the conservative treatment of the pulp after cocaine cataphoresis. The indications are that the pulps capped after the use of this means of obtunding have given better results, as evidenced by the greater absence of subjective indications of hyperæmia than where capped under ordinary circumstances.

surface of the gold was varnished; the edges next the cavity could not be so treated. Cocaine hydrochlor. eighteen per cent.; fifteen cells; twenty-third pin; twelve minutes; relief nearly complete, there appearing some sensation near the gold.

CASE VI.—H. H. F.; right superior second bicuspid; proximate; cocaine citrate twenty-four per cent.; fifteen cells; fifty-ninth pin; ampèrage five-tenths millii; twelve minutes; relief complete.

CASE VII.—H. H. F.; distal left superior first bicuspid; cocaine citrate twenty-four per cent.; twenty-eighth pin; fifteen minutes; relief nearly complete; pulp found to be exposed and conservatively treated.

CASE VIII.—M. N.; mesial left superior first molar; cocaine citrate; fifteen cells; thirty-fifth pin; sixteen minutes; relief nearly complete, a little sensibility appearing at the close of the grooving.

CASE IX.—H. H. F.; right superior second molar occlusal; fifteen cells; fifty-sixth pin; eight minutes; relief complete.

CASE X.—Miss E. P.; distal left inferior first molar, extremely sensitive; cocaine citrate twenty-four per cent.; fifteen cells; twenty-eighth pin; fourteen minutes; relief complete.

CASE XI.—Mrs. P. W. E.; left superior cuspid, proximal, first application; ten cells; thirteen minutes; second application, fifteen cells; thirty-seventh pin; ten minutes; ampèrage, one-tenth millii; relief complete.

CASE XII.—Miss M.; mesial right superior bicuspid; fifteen cells; nineteenth pin; twenty-two minutes; relief complete; pulp exposed.

CASE XIII.—Mrs. S. S. M.; right inferior second bicuspid; pulp actively congested. On passing drill through buccal enamel found dentine acutely sensitive. In a moment after this was obliged to withdraw drill. Applied cocaine citrate; fifteen cells; forty-second pin; twelve minutes. The drill was then carried forward without pain, the patient not perceiving when the pulp was encroached upon. It bled freely; applied arsenous acid.

CASE XIV.—Mrs. F. C. W.; distal right superior second bicuspid; cocaine hydrochlor.; fifteen cells; fifty-sixth pin; thirteen minutes; relief complete.

CASE XV.—Mr. J. C. H.; left superior third molar; buccal surface; fifteen cells; sixty-sixth pin; twelve minutes; relief partial. Here, on account of the position, insulation of the tooth could not be secured.

CASE XVI.—Continuation of treatment of Case XIII., four days subsequently. Pulp devitalized half distance; sensibility excessive; applied cocaine citrate and also the hydrochloride by cataphoresis, without the least impression; also instilled cocaine, which proved equally valueless.

CASE XVII.—Mrs. F. C. W.; mesial right inferior first molar; fifteen cells; thirty-eighth pin; ampèrage, three-tenths millii; relief nearly complete.

CASE XVIII.—W. M. C.; distal right superior first molar; fifteen cells; thirty-fifth pin; ampèrage, four-tenths millii; thirteen minutes; relief complete. In this case there was no electrical irritation until the fourteenth pin had been passed; cavity broad and shallow.

CASE XIX.—Master A. C.; distal left superior first molar; cocaine hydrochlor. twenty-four per cent.; fifteen cells; forty-second pin; eight minutes; relief complete.

CASE XX.—Mrs. F. C. W.; mesial left superior third molar; cocaine citrate twenty-four per cent.; fifteen cells; forty-fifth pin; ampèrage, eight-tenths millii; relief not complete, consequent upon imperfect insulation as evidenced by the ampèrage.

CASE XXI.—Master M. E. B.; mesial right superior first molar; cocaine citrate twenty-four per cent.; fifteen cells; forty-seventh pin; ampèrage, three-tenths millii; twelve minutes; relief not complete; second application, twenty cells; seventieth pin; five minutes; relief complete.

CASE XXII.—Mrs. F. C. W.; mesial right superior second molar; cocaine citrate; twenty cells; thirtieth pin; eighteen minutes; relief complete, except at occlusal margin.

CASE XXIII.—Mrs. T. M. S.; distal left inferior second molar; cocaine citrate twenty-four per cent.; fifteen cells; forty-eighth pin; ampèrage, four-tenths millii; eleven minutes; no relief.

CASE XXIV.—Same patient and same cavity, three days after first application; cocaine citrate; fifteen cells; twentieth pin; twenty minutes; no relief. Patient very sensitive to electrical irritation.

CASE XXV.—B. C.; distal left superior first bicuspid and mesial left superior second bicuspid; fifteen cells; thirtieth pin; relief only partial.

CASE XXVI.—Mr. H. H.; distal left inferior second bicuspid; cocaine hydrochlor. twenty-four per cent.; fifteen cells; fifty-sixth pin; eleven minutes; relief complete.

CASE XXVII.—Miss L. N.; distal second molar; cocaine hydrochlor. twenty-four per cent.; 110-volt Edison current, with Flemming's controller carried through one-fourth of the circuit; twelve minutes; relief nearly complete.

CASE XXVIII.—Miss E. C.; mesial left superior lateral; fifteen cells; thirty-sixth pin; thirteen minutes; relief complete.

CASE XXIX.—Master H. Du P.; mesial right superior central; cocaine hydrochlor. twenty-four per cent.; ten cells; fortieth pin; thirteen minutes; relief almost complete

CASE XXX.—Same patient; distal right superior cuspid; cocaine hydrochlor. twenty-four per cent.; ten cells; fortieth pin; twelve minutes; relief complete.

CASE XXXI.—Master W. S. G.; distal right superior central; cocaine hydrochlor.; ten cells; thirty-fifth pin; ampèrage, one-tenth millii; fourteen minutes; relief complete. For this patient it had previously been necessary to administer ether to permit the preparation of carious cavity.

CASE XXXII.—Mr. H. H.; distal left superior central and mesial of left superior lateral; cocaine hydrochlor. twenty-four per cent.; fifteen cells; forty-second pin; ampèrage, one-tenth millii; twenty-four minutes; relief of central complete. In the final cutting of the lateral, some pain.

CASE XXXIII.—Same patient; mesial of both superior centrals; cocaine hydrochlor. twenty-four per cent.; fifteen cells; fifty-sixth pin; ampèrage, one-tenth millii; twenty-four minutes; relief nearly complete. In these cases the sensibility was of unusual acuteness.

CASE XXXIV.—W. McG.; mesial right inferior first molar; cocaine hydrochlor.; fifteen cells; fifty-sixth pin; ampèrage, four-tenths; twelve minutes; relief complete. Pulp found exposed and conservatively treated.

CASE XXXV.—Miss M. N.; mesial left superior first molar; cocaine hydrochlor. twenty-four per cent.; fifteen cells; ampèrage, four-tenths millii; fifteen minutes; relief nearly complete. Slightly sensitive at inner margin.

CASE XXXVI.—Same patient; right inferior second bicuspid; cocaine hydrochlor.; fifteen cells; fifty-sixth pin; ampèrage, seven-twentieths millii; nine minutes; relief nearly complete.

CASE XXXVII.—Same patient; distal left inferior second bicuspid; cocaine hydrochlor.; fifteen cells; fiftieth pin; ampèrage, three-tenths millii; thirteen minutes; relief complete.

CASE XXXVIII.—Mrs. A. J. D.; distal left superior lateral; cocaine hydrochlor.; fifteen cells; thirty-sixth pin; ampèrage, two-tenths millii; thirteen minutes; relief complete, except at the occlusal margin.

CASE XXXIX.—Miss A. H. H.; mesial left superior first bicuspid; cocaine hydrochlor.; fifteen cells; forty-eighth pin; ampèrage, two-tenths millii; seven minutes; relief absolute. At the close of the application the patient fainted, manifesting considerable cyanosis. The syncope was probably due to nervous impression caused by the result of a previous medical administration of electrical current, when the same condition ensued. During the application there was no electrical irritation of the tooth.

CASE XL.—Mrs. J. H. P.; mesial left inferior third molar; caries extending below the gum; pulp evidently exposed and dentine extremely sensitive; insulation imperfect; cocaine hydrochlor. twenty-four per cent.; fifteen cells; twenty-third pin; ampèrage, four-tenths; thirteen minutes; relief not complete, but sufficiently so to permit removal of caries and the uncovering of the pulp.

It were well to state that in some instances, where the relief did not appear absolute, the results would probably have been more satisfactory had the current been longer continued.

